# Evaluation of Therapeutic Intervention with a Natural Product in Cutaneous Wound Healing: The Use of Capybara Oil

**DOI:** 10.1155/2013/217198

**Published:** 2013-06-10

**Authors:** Polyana Cury Marinho, Rodrigo Neto-Ferreira, Jorge José de Carvalho

**Affiliations:** Laboratory of Ultrastructure and Tecidual Biology, Biomedical Center, Institute of Biology, State University of Rio de Janeiro, 20551-030 Rio de Janeiro, RJ, Brazil

## Abstract

Capybara oil is commonly used for cutaneous wound healing in traditional South American medicine, although its beneficial effect has never been experimentally proven. The aim of this study was to investigate the effects of the topical application of capybara oil on skin wounds in Swiss mice. The following characteristics of the wounds were observed and evaluated: wound contraction and reepithelialization, the number of polymorphonuclear leukocytes and mast cells, the thickness of the neoepidermis, and the distribution of collagen and elastic fibers. Our study showed that oil extracted from subcutaneous capybara fat was beneficial for wound healing, indicating that capybara oil plays an important role in promoting tissue repair.

## 1. Introduction

Cutaneous wound healing is a complex process involving a series of sequential and overlapping phases, including inflammation, proliferation, and remodeling. This process requires interactions between a variety of cell types, multiple cytokines, growth factors, and extracellular matrix (ECM) molecules [1]. Based on traditional medicine, studies have shown improvement in the healing process with the use of natural products [2], including vegetable [3–5] and animal oils [2, 6–8]. Fatty acids (FAs) from these oils can modulate events such as inflammation, cell migration, angiogenesis, and ECM remodeling [5], and they can also act as chemotactic agents for leukocytes and promote cell proliferation [9, 10]. It is known that moist dressing oil, a typical application of FAs, acts as a protective barrier against microorganisms, prevents tissue dehydration, and maintains local body temperature [9]. The capybara (*Hydrochaeris hydrochaeris*) is the largest rodent in existence and is widely distributed in South America, being the most wild native animal raised in Brazil. It is a good economic resource because of its hide, meat, and oil. The study of oil refers to how best to utilize the byproducts of capybara, since the consumption of their meat is growing every day in Brazil, having good market acceptance [11]. Oil extracted from capybara fat contains 19.6% linolenic, 17.9%  *α*-linoleic [12], 39.8% oleic, and 20.7% palmitic [13] FAs. It has been shown that this oil has certain properties, it is effective in lowering cholesterol levels in hypercholesterolemic rats, it is used as an oil in a traditional remedy in Paraguay [12], it is used as a tonic for children (Capivarol), it helps to alleviate the symptoms of respiratory diseases: bronchitis and asthma, and it helps in rheumatism. This oil also acts as a powerful agent to promote external wound healing, being used by the people of various Brazilian regions, but this finding has not been experimentally proven in a clinical or laboratory setting [13, 14], making it important to analyze the real effects of oil for later rational use.

## 2. Materials and Methods

### 2.1. Capybara Oil Material

Subcutaneous capybara fat was donated by the “Frigorífico Cerrado Carnes de Caça” in the State of Sao Paulo. Animals were bred in captivity as authorized by the “Brazilian Institute of Environment and Renewable Natural Resources” (IBAMA). Fat was extracted by hand using hydrothermal processing in a water bath. This process yielded oil in a liquid form, which was applied to the lesions.

### 2.2. Animals and Cutaneous Wound Procedure

Forty-two male Swiss mice, which weighed approximately 30 g each, were used for the study. They were individually housed and maintained in standard housing conditions (21 ± 2°C, humidity 60% ± 10%, and 12 : 1 dark-light cycle), and they received water and rodent chow (Nuvilab, Parana, Brazil) *ad libitum. *All procedures were approved by the local Ethics Committee for the Use and Care of Experimental Animals. For wounding, all animals were anaesthetized with an intraperitoneal injection of ketamine (5 mg/kg) and xylazine (2 mg/kg), the dorsal surface was shaved, and a full-thickness excisional wound (0.6 mm^2^) was made on the back of each mouse by removing the skin (epidermis and dermis) and exposing the paniculus carnosus. The wound was allowed to heal by second intension. The discomfort induced by this wound protocol was minimal; thus, no analgesics were required. Animals were separated into two groups: the control group (*n* = 28), which received no treatment, and the capybara oil group (*n* = 28), which received daily topical applications of capybara oil (0.1 mL). The groups were subdivided into four subgroups (*n* = 7) according to the protocol established for macro- and microscopic evaluation of wounds at days 3, 7, 14, and 21 in the postoperatory (PO) period.

### 2.3. Macroscopic Analysis of the Wounded Area

**Figure 1:**
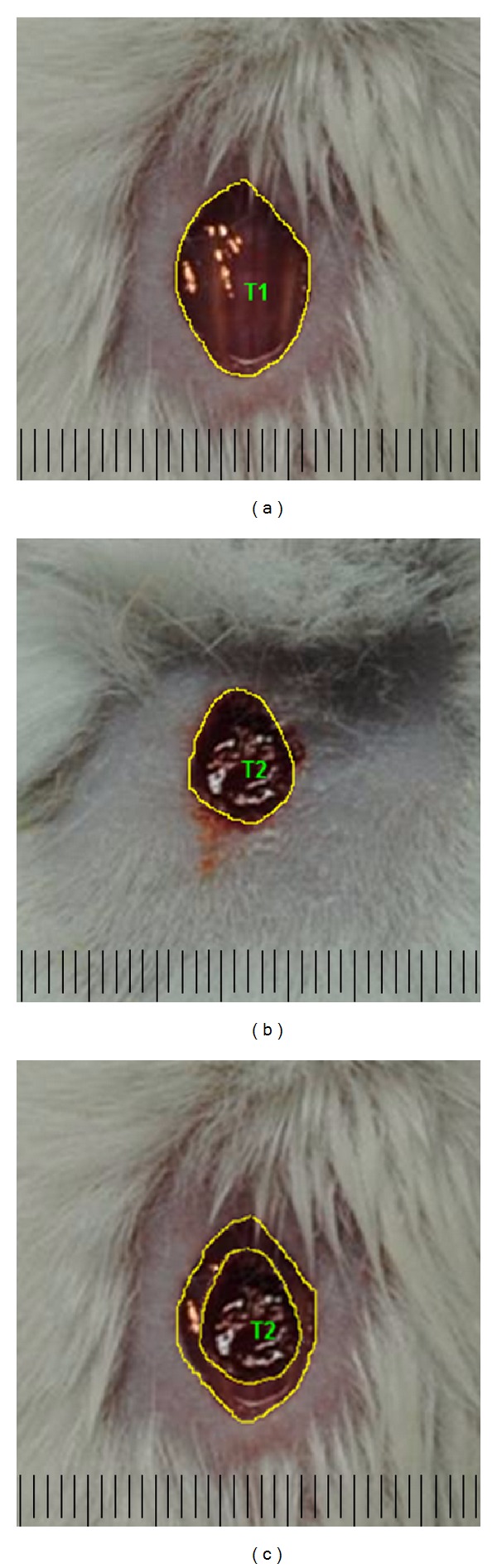
Evaluation of wound contraction and reepithelialization. Photo from (a) total—T1; (b) nonreepithelialized (still uncovered by a new epithelium)—T2; and (c) difference between the two areas—T1 less T2. Bar indicates 1 cm.

The wounds were observed macroscopically during the PO days to evaluate the wound and to verify the presence or absence of secretion and crust. To evaluate the wound contraction and reepithelialization, the lesion margin of each animal, which was represented by an increased area of redness, was traced on a transparent plastic sheet placed over the lesion. Lesion tracings were digitalized, and the wounded area was evaluated using Image-Prow Plus 5.0 (Media Cybernetics, Silver Spring, MD, USA). Wound contraction and reepithelialization were estimated by calculating the difference between the total area of the lesion on the first day of the experiment and the area of the wound that remained uncovered with new epithelium (Figure 1) on PO day 7. Wound contraction data are expressed as the percentage of the area of the contracted wound, and reepithelialization data are expressed as a percentage of the area of the reepithelialized wound at each measurement (mean and standard error).

### 2.4. Tissue Harvesting and Staining

Mice were sacrificed with deep anesthesia (intraperitoneal with sodium pentobarbital, 15 mg/Kg) on PO days 3, 7, 14, and 21. The wound and normal adjacent skin were removed, fixed with a fixative solution (freshly prepared 4% formaldehyde in phosphate buffer at pH 7.2), and embedded in paraplast plus (Sigma-Aldrich, St. Louis, USA). Five micrometer (*μ*m) sections were stained with hematoxylin eosin (HE) to analyze general aspects of the wound, the presence of polymorphonuclear (PMN) leukocytes, and the neoepidermis thickness. Toluidine blue was used for mast cell evaluation; picrosirius red was used for collagen fiber analysis, and Weigert's resorcin-fuchsin was used to identify the fibers of the microfibril-elastin system. For assessment, a videomicroscopic system (Axiolab ZEISS light microscope, HBO 50A/C video-camera and Philips monitor, and Olympus BX53 light microscope, Olympus DP72 video-camera) was used.

### 2.5. Morphometric Analysis

**Figure 2:**
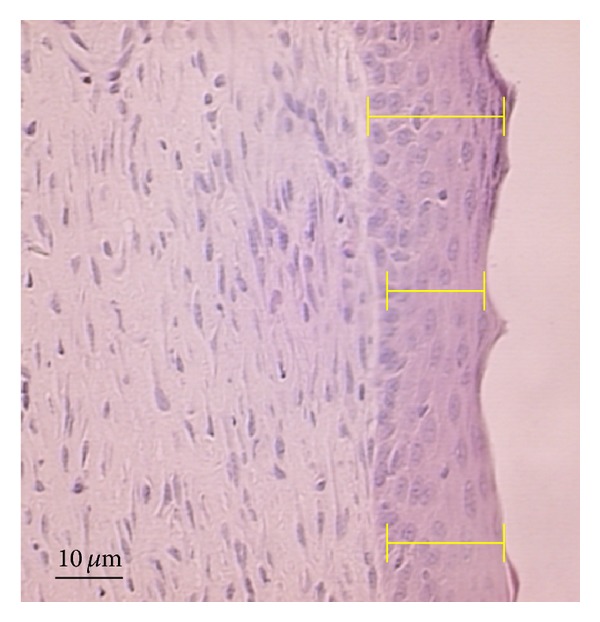
Evaluation of neoepidermis thickness. Photomicrograph of the measurement of the neoepidermis, from three random fields of the dermoepidermal junction to the outermost portion of the granular layer.

Neoepidermis thickness in HE-stained sections was measured in photomicrographs obtained by the videomicroscopic system and evaluated using Image-Prow Plus 5.0. The thickness was measured from the dermoepidermal junction to the outermost portion of the granular layer, as shown in Figure 2. Three random fields per animal were analyzed using the 20x objective, with three measurements in each field. Data are expressed as the mean of the measurements in each wound.

### 2.6. Stereological Analysis

Photomicrographs of the wounds were analyzed to estimate the numerical density per area (*Q*_*A*_) [15] of PMN leucocytes, mast cells, and PCNA-positive cell nuclei in the granulation tissue. The *Q*_*A*_ is defined as the numerical profile of cells within a known test area (expressed as cells/*μ*m^2^) of 3305 *μ*m^2^. Several photomicrographs per group were evaluated, and all cells within the area were counted, except those that touched the borderline (i.e., the forbidden line). For assessment, a videomicroscopic system that displayed photomicrographs on the monitor was used and was calibrated to the micrometer.

### 2.7. Polymorphonuclear Leukocytes and Mast Cells

To evaluate inflammatory infiltration, the number of PMN leukocytes was evaluated in HE-stained tissue sections and quantified in 10 random fields (3305 *μ*m^2^) per animal, using a 40x objective. Results are expressed as the mean number of cells per field. The number of mast cells was evaluated in the toluidine blue-stained tissue sections. Mast cells were counted in 15 random fields (3305 *μ*m^2^) per animal, from the granulation tissue using a 40x objective. The results are expressed as the mean number of cells per field.

### 2.8. Immunochemistry and Quantification

For immunochemistry of proliferating cells, a mouse monoclonal antibody against proliferating cell nuclear antigen (PCNA) (Santa Cruz Biotechnology Inc., Santa Cruz, CA, USA) was used at a 1 : 300 dilution. Sections were incubated in 3% H_2_O_2_ in distilled water to block endogenous peroxidase then in citrate buffer (pH 6.0) to promote antigen retrieval and finally with primary antibody. To detect the primary antibody, a biotinylated secondary antibody (DAKO, Carpinteria, CA, USA) and streptavidin-horseradish peroxidase were used (DAKO). Diaminobenzidine (DAB) was used as a chromogen. Sections were counterstained with hematoxylin. No labeling was observed on the sections in which the primary antibody was omitted (negative control). Cell proliferation was evaluated separately in the neoepidermis and the granulation tissue by counting the number of PCNA-positive cell nuclei. In the neoepidermis for each animal, all basal cells were counted with a 40x objective, and the results are expressed as the percentage of PCNA-positive epithelial cells. In the granulation tissue, ten random fields (3305 *μ*m^2^) per animal were counted with a 40x objective, and the data are expressed as the mean and standard error. For all counts, the videomicroscopic system was used. 

### 2.9. Qualitative Evaluation of Collagen Fibers and the Microfibril-Elastin System

Tissue sections stained with picrosirius red were observed under a polarizing light microscope (Olympus BX53 light microscope and Olympus DP72 video-camera). Photomicrographs were captured for the analysis of collagen fibers in the wounded area. The picrosirius red method highlights the birefringence of normal collagen fibers; thick fibers appear strongly birefringent and yellow-to-red in color, whereas thin collagen fibers are weakly birefringent and greenish [16]. Fibers of the microfibril-elastin system were stained with Weigert's resorcin fuchsin, and for the analysis of elastic fibers in the wounded area, several photomicrographs were observed and captured by a videomicroscopic system.

### 2.10. Data Analysis

All data are presented as the mean ± standard error (mean ± SEM). All statistical analyses were performed by comparing the control group to each treatment group. Data concerning wound contraction, reepithelialization, neoepidermis thickness, PMN leukocyte and mast cell quantification, and the number of PCNA-positive cells were analyzed with a parametric Student's *t*-test with Welch's correction (unpaired test). Values of *P* < 0.05 were considered statistically significant. Statistical analysis was performed using the software GraphPad Prism version 5.0 (GraphPad Software Inc., San Diego, CA, USA).

## 3. Results

### 3.1. Wounded Area

**Figure 3:**
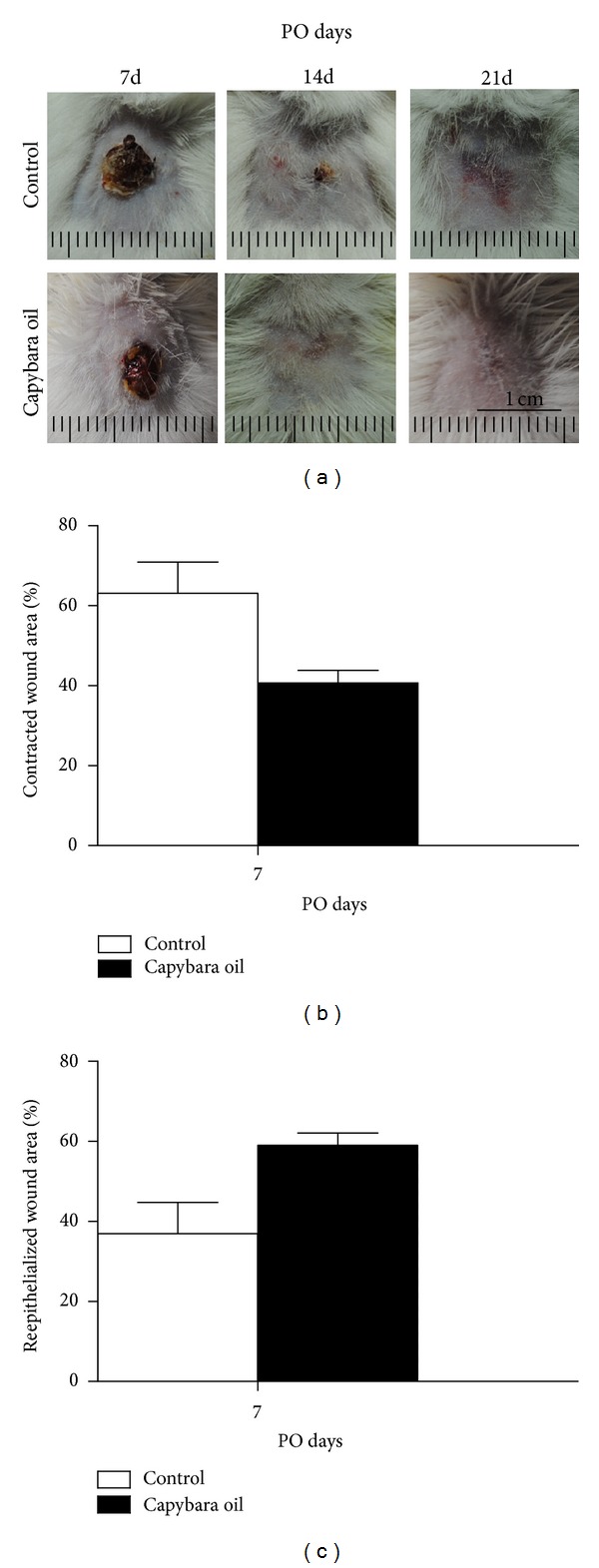
Photo from the macroscopic evaluation of the wounds in the control and the capybara oil groups on PO days 7, 14, and 21 (a). Evaluation of the wound contraction (b) and reepithelialized wounded area (c) in the control (□) and the capybara oil (■) groups, at PO day 7. Data are expressed as the percentage of the initial wounded area or reepithelialized wounded area, as the mean ± SEM (*P < 0,05). Bar indicates 1 cm.

Macroscopic evaluation of the wounds was performed on PO days 3, 7, 14, and 21, and no purulent exudate was found in any of the wounded animals. The crusts started to form on PO day 3 in both groups, and they were bulky, irregular, and rough, with reddish or brownish color. On PO day 7, the capybara oil group presented thicker and wetter crust when compared to the control group, in which the crusts were thinner and drier. On PO day 14, the capybara oil group showed complete retraction of the wound, whereas some wounds in the control group showed a small crust. On PO day 21, complete healing was verified in the control group, but granulation tissue could be observed (Figure 3(a)).

The control and capybara oil groups showed a progressive reduction in wounded area during the experiment. When wound contraction was evaluated on PO day 7, the wounded area was 22.4% smaller in the capybara oil group than in the control group, indicating more contraction (Figure 3(b)).

The reepithelialized wounded area was measured on PO day 7. The percentage of the reepithelialized wounded area was 22.06% larger in the capybara oil group relative to the control group (Figure 3(c)).

### 3.2. Neoepidermis Thickness

**Figure 4:**
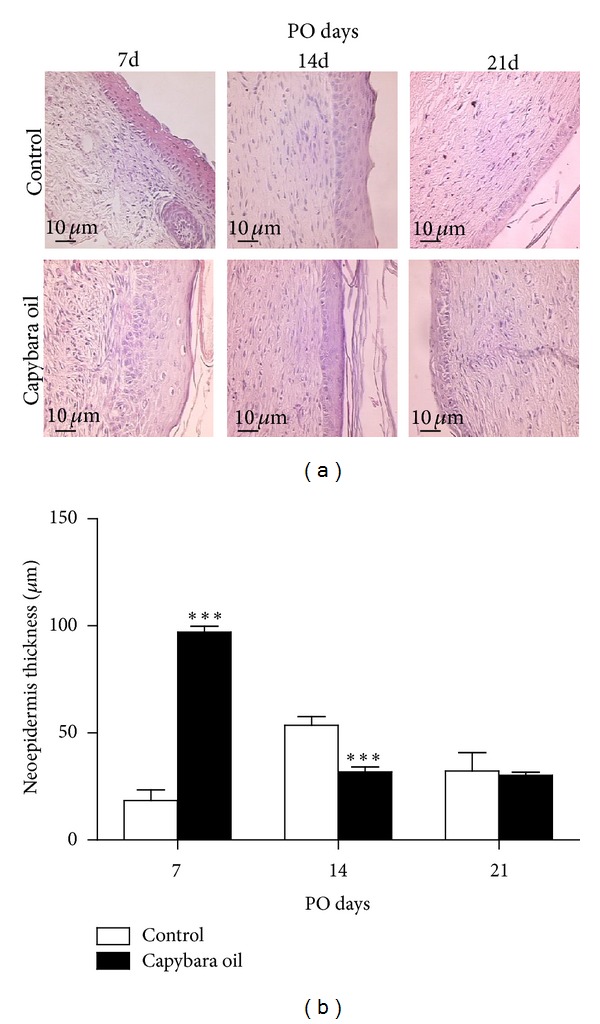
Photomicrograph of the neoepidermis thickness of the wounded area in the control and the capybara oil groups on PO days 7, 14, and 21 (a). Measurement of the neoepidermis thickness in the control (□) and the capybara oil (■) groups on PO days 7, 14, and 21 (b). Data are expressed as the mean ± SEM (***P < 0,0001). Bar indicates 10 *μ*m.

The neoepidermis was evaluated in photomicrographs of the wounds on PO days 7, 14, and 21 (Figure 4(a)). The neoepidermis in the capybara oil group was 78.55% thicker than that in the control group on PO day 7 (Figure 4(b)). In some of the control animals, the neoepidermis was not completely formed. On PO day 14, the neoepidermis in the control group was 21.75% thicker than that in the capybara oil group; there was no significant difference between the groups on PO day 21 (Figure 4(b)).

### 3.3. Polymorphonuclear Leucocytes and Mast Cells

**Figure 5:**
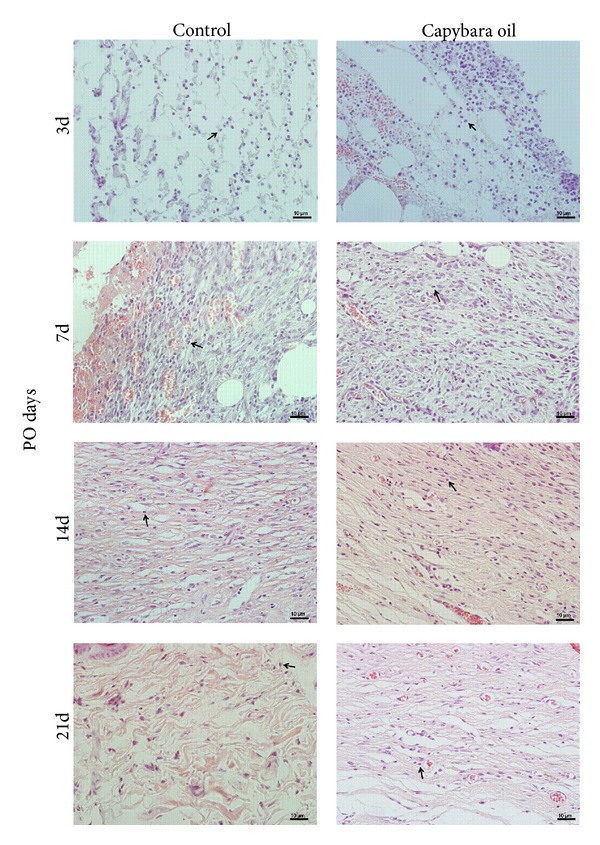
Photomicrograph of PMN leukocytes in the wounded area in the control and the capybara oil groups on PO days 3, 7, 14, and 21. The arrows indicate PMN leukocytes. Bar indicates 10 *μ*m.

**Figure 6:**
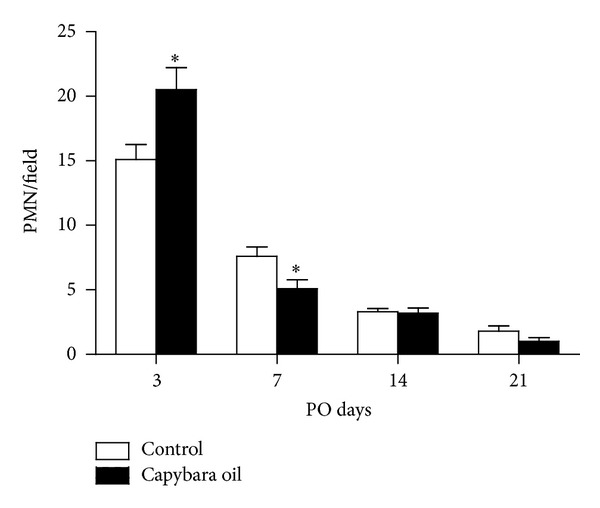
Quantification of PMN leukocytes/field in the wounded area in the control (□) and the capybara oil (■) groups on PO days 3, 7, 14, and 21. Data are expressed as the mean ± SEM (*P < 0,05).

In the photomicrographs, the PMN leukocytes in the wounded area of both the control and the capybara oil groups were quantified on PO days 3, 7, 14, and 21 (Figure 5). Relative to the control group, the number of PMN leucocytes in the capybara oil group was 5.4% higher on PO day 3 and was 2.5% lower on PO day 7; there was no significant difference between the two groups on PO day 14 or 21 (Figure 6). 

**Figure 7:**
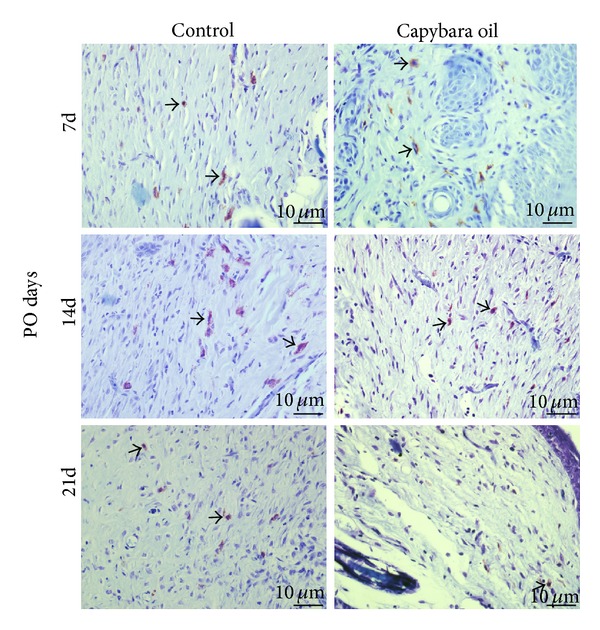
Photomicrograph of mast cells in the wounded area in the control and the capybara oil groups on PO days 7, 14, and 21. The arrows indicate mast cells. Bar indicates 10 *μ*m.

**Figure 8:**
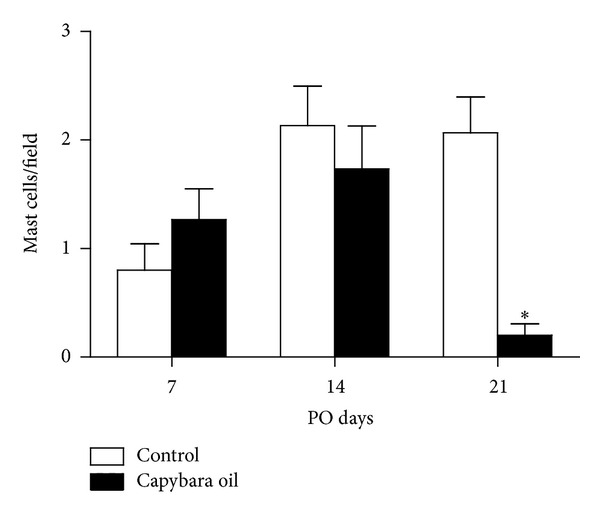
Quantification of mast cells/field in the granulation tissue of the wounded area in the control (□) and the capybara oil (■) groups on PO days 7, 14, and 21. Data are expressed as the mean ± SEM (*P < 0,05).

Quantification of mast cells was performed in the granulation tissue of the wounded area in both the control and the capybara oil groups on PO days 7, 14, and 21 (Figure 7). On PO day 21, the mean number of mast cells was 2.06% higher in the control group relative to the capybara oil group; there was no significant difference between the two groups on PO day 7 or 14 (Figure 8). 

### 3.4. Quantification of Cell Proliferation

The number of PCNA-positive epithelial cells was calculated on PO day 14, and the number of PCNA-positive granulation tissue cells was calculated on PO days 7 and 14. In all groups, PCNA-positive epithelial cells were observed in the migrating tongue and extended to the tip. PCNA-positive granulation tissue cells were homogeneously found in the wounded area. There was no significant difference between the two groups (data not shown).

### 3.5. Collagen Fibers

**Figure 9:**
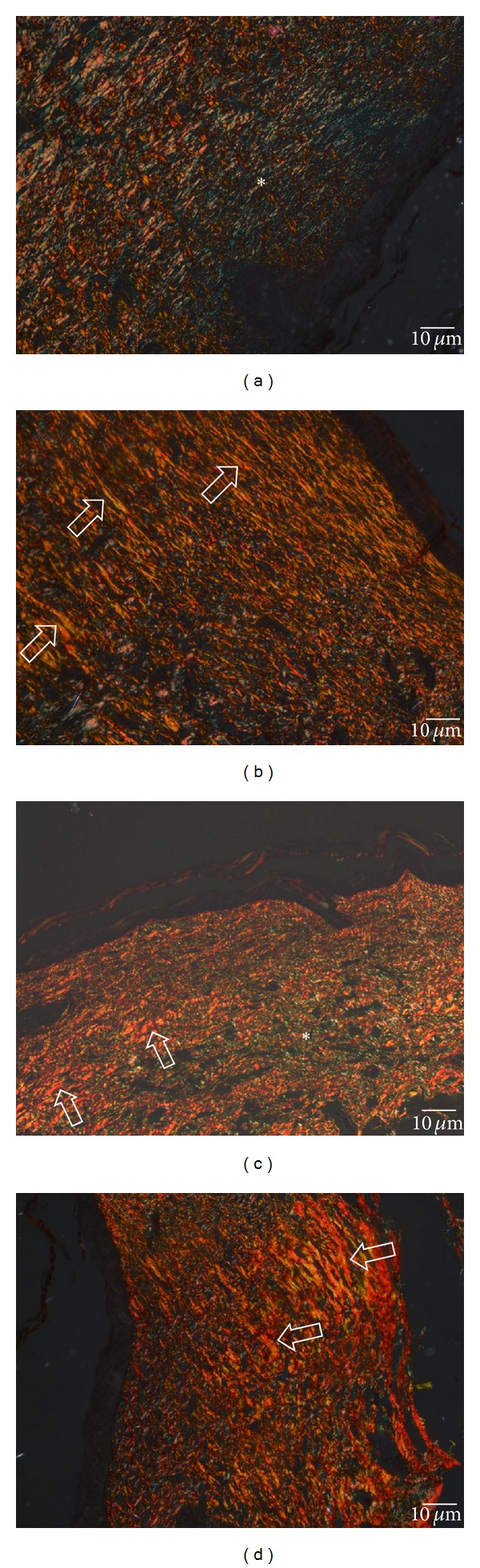
Photomicrograph of collagen deposition and organization in the dermis of the wounded area in the control and capybara oil groups on PO days 14 and 21. On PO day 14, the control group (a) shows a high prevalence of greenish fibers (* ), and the capybara oil group (b) shows a high prevalence of yellow-reddish fibers (open arrow). On PO day 21, the control group (c) shows a few greenish fibers (* ) and yellow-reddish fibers (open arrow), and the capybara oil group (d) shows a prevalence of thick yellow-reddish fibers (open arrow). Picrosirius staining was observed under polarized light. Bar indicates 10 *μ*m.

Collagen fiber deposition and organization were evaluated under polarized light in the picrosirius red-stained sections on PO days 14 and 21. On PO day 14, the wounded area of the control group showed more greenish collagen fibers that were fragmented and had a random arrangement, and there were a few thin yellow-reddish collagen fibers that also had a random arrangement (Figure 9(a)). In contrast, the capybara oil group showed a prevalence of thick yellow-reddish collagen fibers that were elongated and arranged parallel to the surface (Figure 9(b)). On PO day 21, the control group had a few greenish collagen fibers and a few yellow-reddish collagen fibers (Figure 9(c)). Conversely, the capybara oil group showed a larger number of thick yellow-reddish collagen fibers, and the fibers were more organized (Figure 9(d)).

### 3.6. Microfibril-Elastin System

**Figure 10:**
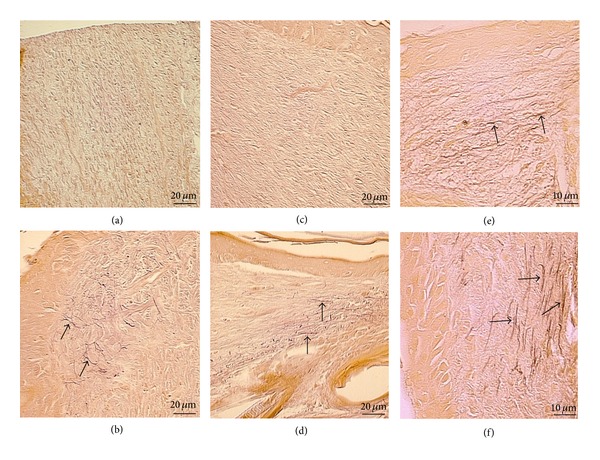
Photomicrograph of elastic fibers in the microfibril-elastin system in the dermis of the wounded area. On PO day 7, elastic fibers were not found in the control group (a), whereas elastic fibers were observed in the capybara oil group (b). On PO day 14, the fibers were not observed in the control group (c), but they were observed in the capybara oil group (d). On PO day 21, elastic fibers were observed in the control (e) and the capybara oil (f) groups. Arrows indicate the elastic fibers. Weigert's resorcin fuchsin staining is shown. Bar indicates 20 *μ*m.

The presence of elastic fibers was evaluated in a microfibril-elastin system with Weigert's resorcin fuchsin-stained sections on PO days 7, 14, and 21. In the capybara oil group, the microfibril-elastin system in the dermis was observed in the wounded area on PO days 7 and 14 (Figures 10(b) and 10(d)), but this system was not found in the control group (Figures 10(a) and 10(c)). On PO day 21, this system was detected in both the control group (Figure 10(e)) and the capybara oil group, but substantially more elongated fibers were found in the capybara oil group (Figure 10(f)). 

## 4. Discussion

This study is the first to show that the application of capybara oil to excisional cutaneous wounds can affect wound healing in Swiss mice. The effect of the capybara oil was evaluated for general aspects of the wounded area, wound contraction, reepithelialization, neoepidermis thickness, cellular proliferation, the amount of PMN leukocytes and mast cells, and the presence of collagen and elastic fibers. The oil accelerated contraction and reepithelialization of the wounded area, microscopically improved reepithelialization by generating a larger neoepidermis, and enhanced remodeling of the ECM by improving collagen deposition and organization as well as early elastogenesis in the wounded area.

Several studies have demonstrated the effect of the topical application of animal oils in the healing process. The topical application of 25% cod liver oil ointment, which is rich in polyunsaturated fatty acids, was shown to accelerate the epithelial and vascular components of healing in mice [6] and to accelerate healing by reducing the wounded area [17]. Moreover, topically applied emu [7] and crocodile [8] oils accelerated the healing of rat burns.

The crust is formed on the surface of the wound when the fibrin clot dehydrates as a result of coming in contact with the environment, and this crust is essential for homeostasis, for temporary closure of the wound, and for controling bacterial contamination. While protecting the wound externally, the crust can negatively affect wound healing in the late stages, impairing reepithelialization and collagen deposition [18]. In the present study, the crusts on the animals in the capybara oil group were thicker and wetter than those in the control group on PO day 7. In these phases, the crust also promoted healing. However, on PO day 14 a few of the control animals still showed some crusts in the superficial wounded area, which may have delayed the healing process.

Wound closure is a combination of wound contraction, mediated by myofibroblasts, and reepithelialization, which is caused by the penetration of the new epithelium into the granulation tissue [1]. According to Cardoso et al. [19], topical application of linoleic and oleic FAs to cutaneous wounds can significantly reduce the wounded area by the fifth day and can cause complete retraction of the wound by the fifteenth day, and application of linolenic FA can cause complete retraction by the seventeenth day. In our study, compared to the control group, the capybara oil group showed greater wound contraction on PO day 7 and complete retraction of the wound by PO day 14. In contrast, the control group showed complete retraction of the wound on PO day 21.

Reepithelialization is the process of restoring an intact epidermis after skin injury, which is accomplished by the formation of new epithelium, also known as neoepidermis formation. Neoepidermis formation consists of the proliferation and subsequent migration of keratinocytes from adjacent skin into the inside of the wound, gradually covering the injured area [1]. In the present study, consistent with the data regarding wound contraction, the application of capybara oil was beneficial for reepithelialization, leading to a larger reepithelialized wounded area in the treated group compared to control group on PO day 7. To evaluate the reepithelialization microscopically, the neoepidermis thickness was measured and was shown to be thicker in the capybara oil group compared to the control group on PO day 7. 

The inflammatory response is a limiting factor in tissue repair after injury. In the early phases, this response is required and beneficial because it induces the production of proinflammatory cytokines, recruits macrophages, stimulates cell proliferation and angiogenesis [20], and promotes debridement and bacterial death during the initial phase of wound healing [1, 21]. However, excessive inflammation causes massive destruction of the wounded area and reduces fibroblast proliferation, ECM synthesis, and angiogenesis [22]. After the topical application of oleic and linoleic FAs to wounds in rats, Pereira et al. [10] reported a significant increase in the number of PMN leucocytes that migrated to the injured area in the initial phase of inflammation. In our study, capybara oil increased the number of PMN leukocytes on PO day 3 and gradually reduced the number in the subsequent PO days.

It is known that mast cells, which are found in conjunctive tissue, are mobilized during wound healing and participate in all phases of the process [23], especially in the inflammatory phase, because these cells are the first to release inflammatory mediators involved in vasodilatation [1, 23] and the recruitment and increased survival of leukocytes [23]. However, it has been shown that prolonged mobilization of mast cells until 21 days after wound formation can damage the formation of granulation tissue [24] and that an excess of mast cell mediators (such as kinases) may delay the migration of epithelial cells [25, 26]. According to Barroso et al. [23], propolis has potential anti-inflammatory effects in acute inflammation and reduces the number of mast cells, but it does not have the same effect in the later stages of repair. In the present study, capybara oil did not affect the number of mast cells in the wounded area on PO day 7 or 14, but the number was reduced on PO day 21 relative to the control group, thus suggesting that the oil has an anti-inflammatory effect late in the healing process.

The use of immunochemistry techniques with monoclonal antibodies was used to recognize antigens expressed in proliferating cells [27]. During reepithelialization, keratinocyte proliferation ensures that there is an adequate supply of cells to migrate into and cover the wound [1]; however, keratinocyte proliferation may occur independently of keratinocyte migration during reepithelialization [16]. Furthermore, during the dermal reconstruction by granulation tissue formation, the fibroblasts proliferate and subsequently migrate to the wounded area and then deposit and organize the ECM [1]. After wound contraction, there is a large number of proliferating cells that promote granulation tissue formation, and this number gradually decreases as the tissue matures [28]. In this study, the similar number of PCNA-positive nuclei in the neoepidermis and granulation tissue in the control and the capybara oil groups suggests that the capybara oil did not affect the proliferation of epithelial cells and fibroblasts. However, because the neoepidermis was thicker in the early stages of healing, it is possible that capybara oil affects the migration of keratinocytes into the wounded area.

Within the interstitial ECM, collagen is the most abundant fibrous protein; collagen constitutes the main structural element of the ECM, provides tensile strength, regulates cell adhesion, supports chemotaxis and migration, and directs tissue development [29]. At least 28 different types of collagen exist in vertebrates [30], but in the skin dermis, type I and type III collagen are the most abundant. During remodeling of the ECM in wound healing, the granulation tissue is rich in type III collagen, and the blood vessels are progressively replaced with type I collagen, forming a collagenous scar that resembles normal skin [1]. Type III collagen appears after 48–72 hours and is secreted for 5 to 7 days [19]. In this study, capybara oil enhanced collagen deposition and organization and showed more mature collagen fibers (thick yellow-reddish fibers with a pattern similar to normal skin) that were well organized in the wounded area, whereas the control group showed a large number of immature collagen fibers (thin greenish fibers) with disturbed organization.

The microfibril-elastin fiber system is composed of two main components: fibrillin and elastin. The relative distribution of these components results in the formation of three types of fibers: oxytalan, elaunin, and elastic fibers [31, 32]. To provide necessary strength and elasticity, the connective tissue of normal skin, similar to that of lungs and blood vessels, has these fibers [33]. The elaunin and elastic fibers correspond to the consecutive stages of normal elastogenesis and, because of their high content of elastin, act by absorbing the shock due to stretching and compression of the dermis where mechanical stress is intense [34]. The microfibril-elastin fiber system can be stained by specific staining methods, including Weigert's resorcin fuchsin, which is a valuable technique for observing these fibers in tissues [32]. However, the elastic fibers are rarely considered in the wound-healing process. Some studies reported in the literature refer to the distribution of elastin and fibrillin in mature and hypertrophic scars and keloids [35]. In our experiment, capybara oil accelerated elastogenesis in the dermis and showed an early emergence of elaunin and elastic fibers in the microfibril-elastin system in the wounded area.

## 5. Conclusion

Our study showed that the topical application of capybara oil can enhance excisional wound healing in a Swiss mouse model. The animals treated with capybara oil demonstrated quantitative and qualitative changes in the epidermal and dermal repair of lesions. Our results indicate an important role of capybara oil in the healing process. These findings expand the information regarding the properties of this oil and can lead to improved therapeutic resources for treating skin wounds.
